# Assessment of the Hyperspectral Data Analysis as a Tool to Diagnose *Xylella fastidiosa* in the Asymptomatic Leaves of Olive Plants

**DOI:** 10.3390/plants10040683

**Published:** 2021-04-01

**Authors:** Carmela Riefolo, Ilaria Antelmi, Annamaria Castrignanò, Sergio Ruggieri, Ciro Galeone, Antonella Belmonte, Maria Rita Muolo, Nicola A. Ranieri, Rossella Labarile, Giovanni Gadaleta, Franco Nigro

**Affiliations:** 1 Research Centre for Agriculture and Environment, Council for Agricultural Research and Economics (CREA-AA), 70125 Bari, Italy; sergio.ruggieri@crea.gov.it; 2 Dipartimento di Scienze del Suolo, della Pianta e degli Alimenti, Università degli Studi Aldo Moro, Via Amendola 165/A, 70126 Bari, Italy; ilaria.antelmi@gmail.com (I.A.); rossella.labarile@uniba.it (R.L.); 3 Department of Engineering and Geology (InGeo), Università degli Studi Gabriele D’Annunzio, Chieti-Pescara, 66013 Chieti, Italy; annamaria.castrignano@unich.it; 4 Water Research Institute, National Research Council (CNR-IRSA), 70125 Bari, Italy; ciro.galeone@ba.irsa.cnr.it; 5 Institute for Electromagnetic Sensing of the Environment, National Research Council (CNR-IREA), 70126 Bari, Italy; belmonte.a@irea.cnr.it; 6 Servizi di Informazione Territoriale S.r.l., 70015 Noci, Italy; mariarita.muolo@sit-puglia.it (M.R.M.); nicola.ranieri@sit-puglia.it (N.A.R.); 7 Independent Researcher, 76011 Bisceglie, Italy; gadaleta73@epap.sicurezzapostale.it

**Keywords:** hyperspectral analysis, *Xylella fastidiosa*, olive plants, real-time PCR, partial least square regression (PLSR), discriminant analysis, unsupervised classification

## Abstract

*Xylella fastidiosa* is a bacterial pathogen affecting many plant species worldwide. Recently, the subspecies *pauca* (*Xfp*) has been reported as the causal agent of a devastating disease on olive trees in the Salento area (Apulia region, southeastern Italy), where centenarian and millenarian plants constitute a great agronomic, economic, and landscape trait, as well as an important cultural heritage. It is, therefore, important to develop diagnostic tools able to detect the disease early, even when infected plants are still asymptomatic, to reduce the infection risk for the surrounding plants. The reference analysis is the quantitative real time-Polymerase-Chain-Reaction (qPCR) of the bacterial DNA. The aim of this work was to assess whether the analysis of hyperspectral data, using different statistical methods, was able to select with sufficient accuracy, which plants to analyze with PCR, to save time and economic resources. The study area was selected in the Municipality of Oria (Brindisi). Partial Least Square Regression (PLSR) and Canonical Discriminant Analysis (CDA) indicated that the most important bands were those related to the chlorophyll function, water, lignin content, as can also be seen from the wilting symptoms in *Xfp*-infected plants. The confusion matrix of CDA showed an overall accuracy of 0.67, but with a better capability to discriminate the infected plants. Finally, an unsupervised classification, using only spectral data, was able to discriminate the infected plants at a very early stage of infection. Then, in phase of testing qPCR should be performed only on the plants predicted as infected from hyperspectral data, thus, saving time and financial resources.

## 1. Introduction

*Xylella fastidiosa* subspecies *pauca* (*Xfp*)*,* a Gram-negative bacterium in the family *Xanthomonadaceae* (γ-proteobacteria), is one of the most dangerous plant pathogens worldwide [1]. It colonizes the xylem of the host, and is transmitted by several xylem sap-feeding insect vectors (Homoptera, Auchenorrhyncha) [2,3]. Formerly restricted to the Americas, a very aggressive genotype, *Xfp* ST53, has been reported in Apulia [4,5] as responsible for the Olive Quick Decline Syndrome (OQDS), a vascular disease causing the death of millions of young and centenarian olive trees [6]. The disease appeared on the west coast of the Salento Peninsula (Apulia region, southeastern Italy) in 2013 and spread rapidly, causing devastating effects on olive trees cultivation, with serious impact on the economy, landscape, environment, and cultural heritage of the region [7]. According to a recent estimation, around 4 million of olive trees have been killed or severely impaired in their productivity in the Salento areas, thus, causing an economic loss of approximately 390M€ [8]. Subsequently, *Xfp* and other subspecies (i.e., *X. f.* subspecies *multiplex*, and *X. f.* subspecies *fastidiosa*) and genotypes, were also detected in insular and mainland France and Spain, as well as in Portugal, thus, spreading in the world’s largest olive-growing area (more than 2.5 million hectares) and threatening the whole Mediterranean agriculture (grapevine, almond, and citrus) [9]. Based on these data, it could be foreseen that for the entire European territory, the risk and the economic losses arising from *Xylella fastidiosa* might be much higher [6].

The localization of the pathogen into the xylematic vessels determines an alteration of the raw sap movement, thus, impairing the water and nutrition supply in the affected twigs and leading to withering and desiccation of scattered shoots and small branches in the canopy. Bacterial cells attach to the vessel wall and, by multiplying, they aggregate in a biofilm matrix that includes nucleic acids, proteins, humic substances, and exopolysaccharide (EPS) protecting bacterial communities [10]. This biofilm is the main factor responsible for the blockage of water movement in *X*. *fastidiosa*-infected plants, even if some studies have found that vessel occlusions are the active host plants responses as defense mechanism [11,12,13,14].

The diagnosis of *X. fastidiosa* is difficult, because the pathogen infects a wide range of wild and cultivated plant species without causing any apparent symptoms for variable length of time. In the case of olive trees, it has been estimated that the asymptomatic period lasts approximately 1.2 years [8,9,10,11,12,13,14,15]. The initial symptom can be observed on the terminal leaf tip of the affected twigs, which turns dark yellow to brown, a condition that spreads inward and leads to complete desiccation. However, the appearance of these symptoms identifies an advanced stage of the infection that is no longer reversible and leads to the death of the plant [16]. After the appearance of the initial symptoms, the desiccation extends to the rest of the canopy and the collapse of the entire tree follows within 3.5–4 years approximately [8], although significant variations may occur, depending on the olive cultivar and the age of the plants [5].

The variation of plant reflectance responses, measured by means of spectral indices in Visible and Near Infrared (NIR) spectral range, has been widely used as an indicator of disease development in the last years, being a feasible and not invasive monitoring means [17,18]. Since a typical symptom of *Xf* infection is the leaf scorching and desiccation, it might be interesting to assess the water stress in the infected olive plants. Sun et al. (2008) [19] studied the effect induced by a water stress on the reflectance spectrum response of olive trees. As a result, they found that 16 relevant wavelengths were responsive: (1) 524 nm, 555 nm, 572 nm, and 673 nm in visible region; 715 nm, 919 nm, 958 nm, and 1098 nm in NIR region; 1140 nm, 1202 nm, 1225 nm, 1272 nm, 1391 nm, 1455 nm, 1502 nm, and 1656 nm in the shortwave infrared region (SWIR). They developed an algorithm, the Relative Reflectance Increment (RRI), able to quantify and compare the change of reflectance spectra at different reference wavelengths in response to abiotic water stress.

Zarco-Tejada et al. (2018) [20] compared Support Vector Machine (SVM), neural networks, and Linear Discriminant Analysis (LDA) procedures for detecting infected plants. They classified olive trees: in the field, scoring visually the plants as symptomatic and asymptomatic, as well as in the laboratory, determining the DNA of *Xfp* by quantitative Polymerase Chain Reaction (qPCR) and rating the plants as infected and uninfected. As a result, they found that the Normalized Phaeophytinization Index (NPQI) [21,22] measured by remote sensing on board of aircraft, together with Crop Water Stress Index (CWSI), and the thermal-based stress trait best distinguished *X. fastidiosa* symptomatic from asymptomatic trees. The accuracy of disease detection was confirmed by qPCR and exceeded the 80% when high-resolution fluorescence and thermal stress indicators were coupled with photosynthetic traits sensitive to rapid pigment dynamics and degradation.

Therefore, considering the relevant socio-cultural and economic impact of *Xfp* on olive cultivation, as well as the infectiousness and the destructiveness of the disease, the monitoring of the infected area must be fast and reliable, thus, allowing an early diagnosis of the disease also when the symptoms are not yet visible. In fact, any delay would preclude the effectiveness of the mandatory phytosanitary measures to slow down the epidemic progression (i.e., felling of the infected trees), thus, increasing the infection risk for the surrounding plants. Considering the recent developments of hyperspectral spectrometry for the detection of *Xfp*-infected plants, the objectives of this work were to select specific wavelengths, to assess the potential of hyperspectral data analyzed with a proper statistical approach and to discriminate, with a sufficient degree of accuracy, between uninfected and infected but asymptomatic olive leaves. Therefore, olive leaves collected from twigs tested for the presence/absence of the pathogen were analyzed with a hyperspectral apparatus, and then submitted to qPCR analysis. An integrated approach was used, including different statistical techniques: Variable Importance in the Projection (VIP) statistic determination with Partial Least Square (PLS) regression (PLS-VIP), Canonical Discriminant Analysis (CDA)*,* stepwise selection, and unsupervised classification, each one adding an extra piece in the process of extracting information from hyperspectral data related to the infection by *Xfp*.

## 2. Materials and Methods

### 2.1. Site Description

**Figure 1:**
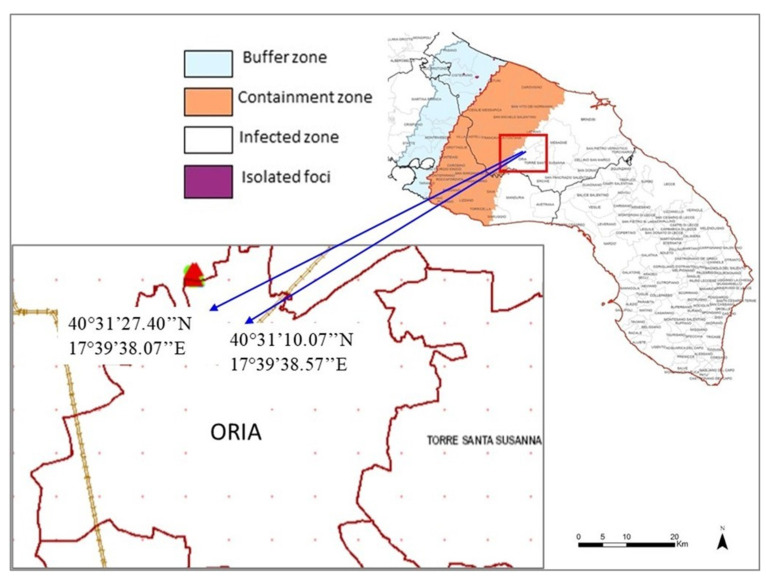
Experimental fields located at Oria (Apulia, province of Brindisi) on the edge between the infected and containment areas. The image was redrawn 1 October 2018 from https://www.emergenzaxylella.it.

Three demarcated areas were identified in Apulia by the Phytosanitary Authority, in order to deal with the diffusion of *Xfp* disease in southern Apulia, and according to the EU Decision 2015/789 and subsequent modifications: (1) the infected zone, the southernmost area of the Salento peninsula where the disease had been diagnosed initially; (2) the containment zone, comprising the last 20 km toward North of the infected zone, where the eradication of the pathogen was mandatory; (3) the buffer zone, 10 Km wide strip after the containment zone, not yet contaminated by *Xfp* (Figure 1). Two experimental fields were selected in the Municipality of Oria (Brindisi), located on the edge between the infected and the containment zones at the time of survey (Figure 1).

### 2.2. Diagnostic Assessment of Plants and Evaluation of Their Phytosanitary Status

The survey was carried out in March 2019. The experimental fields were commercial olive orchards (cv. Cellina di Nardò, approximately 50 years old) located at Oria (province of Brindisi, southeastern Italy). Twenty-five plants were selected in the same proportions of infected (30%) and uninfected (70%) trees present in the experimental fields in a previous survey of October 2018. Before the survey of March 2019, the phytosanitary status of the selected plants was carefully tested, to exclude the occurrence of pathogens causing vascular disease or leaf desiccation other than *Xfp*. The occurrence of vascular fungal pathogens was ascertained by isolation on Potato Dextrose Agar (PDA) amended with antibiotics (500 mgL^−1^, streptomycin sulphate, Sigma-Aldrich). Woody samples (twigs and branchlets) from the selected trees were superficially disinfected with ethanol (80%), then peeled to expose the xylem tissue and examined for the presence of wood discoloration. Subsequently, 10 small wood chips (2 × 2 mm) per sample were seeded onto PDA plates. The developing colonies were checked after 7–14 days incubation at 23 ± 1 °C in dark and transferred in purity to obtain monoconidial isolates. Subsequently, colonies were identified based on their morphological and molecular characteristics.

Three semi-lignified twigs containing at least six mature and asymptomatic leaves, were selected and identified on each monitored plant, to verify without any reasonable doubt, the occurrence of *Xfp* infection. The day before the spectral analysis, two leaves per twig were collected and used for a quantitative measurement of the bacterial DNA in the tissues by qPCR [23,24]. The leaves were collected with scissors mounted on a telescopic rod, closed in a double plastic bag, and transported to the laboratory where they were stored at 5 °C, to be analyzed within 24 hours from the collection. Leaves were disinfected by dipping for 1 min in an aqueous ethanol solution (70%), and then rinsed in sterile distilled water. For each couple of disinfected leaves, midribs portion (approximately 0.1 g) was used to extract Total Nucleic Acid (TNA), following a standard CTAB-based procedure [25]. Aliquots of 2.0 μL of TNA were tested in triplicate with quantitative real-time PCR (qPCR) in 25.0 μL of final reaction volume, according to the protocol of Harper et al. (2010) [26] and using a CFX96 Touch Real-Time PCR apparatus (Biorad, Hercules, CA, USA). As positive control, DNA from a pure culture of *X. fastidiosa* subspecies *pauca* ST53, growing on buffered charcoal yeast-extract agarized medium (BCYE), was used [15]. *Xfp* level occurring in the tested leaves, expressed as the concentration of the bacterial DNA (ng µL^−1^), was determined from the standard curve of a serial dilution (10−10^−7^ ng µL^−1^) of pure bacterial DNA. The genomic DNA quality and quantity were determined using a Nanodrop 2000 spectrophotometer (Thermo Fisher Scientific Inc., Wilmington, DE, USA) and a Qubit 2.0 fluorimeter (Life Technologies Ltd., Paisley, UK). For values of cycles threshold (Ct) ≥ 38, twigs were considered negative.

The day of the spectral analysis, one leaf was randomly chosen on each selected twig and the spectral analysis was performed in field. After the test, the three leaves per tree (one per twig) were pooled and used in laboratory for the detection of *Xfp* by qPCR as specified above [25,26].

Data from the qPCR measurements were subjected to different elaborations, depending on whether they were considered as continuous or binary numerical (0 negative/1 positive) value. In the first case, data were expressed as ng µL^−1^ of bacterial DNA; in the latter, the value 0 was associated with a DNA amount ≤ the detection limit, so declaring the leaves as negative or uninfected; conversely, 1 was attributed to DNA amount > the detection limit, the leaves were considered as positive or infected.

Each olive tree used in the test was defined as “asymptomatic” when it resulted negative or positive for the *Xfp*, but neither desiccated leaves nor fungal pathogens (i.e., *Verticillium dahliae*, *Phaeoacremonium* spp., *Pseudophaeomoniella* spp., *Pleurostomophora richardsiae*, *Neofusicoccum* spp.) were found following both the visual inspection and the isolation procedure.

### 2.3. Spectral Analysis

Leaf spectral measurements were performed in the field with Field Spec IV spectroradiometer (Analytical Spectral Devices Inc., Boulder, CO, USA) using artificial light able to detect a spectral signature in a range of 350–2500 nm. The instrument was equipped with three spectrometers: one for the 350–1000 nm region characterized by a sampling interval of 1.4 nm, the second for 1000–1800 nm region and the third for 1800–2500 nm, these last two with a sampling interval of 2 nm.

Field Spec IV provided spectra with 2151 bands having a resolution of 1 nm. The spectral reflectance data were then averaged over 10-nm intervals, to reduce the number of wavelengths from 2151 to 215, to smooth the spectra and to keep down the risk of over-fitting [27].

A specifically developed leaf clip covering a spot of 10 mm diameter was related to the fiber-optic of spectroradiometer to a halogen lamp 6.5 W, as a light source. In this way, the influence of the atmosphere with the measurements was avoided.

The values of radiance were transformed into spectral reflectance as the ratio between the radiance reflected by the plant and the one from a standard white reference 10 × 10 mm^2^ disk (Spectralon panel, Lab-sphere, Inc., North Sutton, NH, USA). The calibration was repeated for each tree, thus, increasing the comparability of measurements. One measurement on a spot of 10-mm diameter was replicated three times on the leaf selected for each twig: nine individual spectra were collected for each tree and classified with qPCR performed on the pooled sample of the three leaves (as described before). Then 75 leaves were analyzed with the spectroradiometer (three for each of the 25 trees) for a total of 225 spectra collected. The spectra were analyzed individually rather than averaged at leaf level, because the objective of this work was to assess if the methodology provided by hyperspectral analysis can be useful to monitor the presence of Xylella infection.

### 2.4. Pre-Processing Methods

Some spectral pre-processing methods were required to reduce the impact of multiplicative and additive effects of eventual backscattering inside the instrument and to achieve a sufficient degree of accuracy for the prediction models [28].

In this regard, two pretreatments were carried out on reflectance spectra: (1) multiple scatter correction (MSC); and (2) smoothing/denoising with Savitzky–Golay polynomials [29].

Multiplicative scatter correction (MSC) works primarily when the scatter effect is the dominant source of variability and removes additive and multiplicative components. MSC simplifies the calibration model reducing the number of components needed and may improve the linearity between prediction and predictors [30].

Savitzky–Golay (SG) first-order polynomial algorithm reduces the random noise of the measurements. The algorithm uses a moving polynomial fit of any order and the size of the filter consists of (2*n* + 1) points, where n is the half-width of the smoothing window (*w*). The polynomial fit interpolates the points between the 2*n*’s. A window size (*w*) of 11 (*w* = 2*n* + 1) and the second polynomial order were applied here [31].

Spectral data pre-processing was performed with ParLeS software [32].

When the response variable (DNA content) departed from normal distribution, it was transformed in Gaussian ranks by SAS/RANK procedure: the ranks divided by the total number of observations form values in the range 0 to 1, which were used in subsequent processing. Predictors (spectral data) and response variable (Gaussian rank transformed DNA data) were centered and scaled to have the mean at zero and the variance at 1, to place both on the same relative position to their variation in the data in the process of prediction model estimation.

### 2.5. Prediction Model

Among the available multivariate statistical methods to estimate DNA content, Partial Least Squares Regression (PLSR) [28,33] was chosen, due to the high number of predictors. PLSR is based on decomposition of two sets of variables: the matrix **X** of predictors (matrix *n* x *N***X**, where *n* is the number of observations, 225, and *N***X** is the number of reflectance data at different wavelengths, 215) and the vector **Y** of the response variable (vector *n* x *1* of DNA data). PLSR selects successive orthogonal factors (or latent variables) that maximize the covariance between predictors (**X** reflectance) and response variable (**Y**) and explains most of the variation in both. PLSR decomposes *X* (1) and y (2) into factor scores (*T*) and factor loadings (*P′* and *q*) according to:

(1) $$X=TP^{\prime }+E$$

(2) $$y=Tq+f^{\;}$$

A good PLSR model has few PLSR factors (T) that explain most of the variation in both predictors and responses. The residuals E and f are negligible. Therefore, by combining the two previous equations, the variable y (DNA content) can be estimated as a linear combination of a smaller number of spectra (x), through the estimated loadings [33]. For more details on PLSR method, see, e.g., Martens and Næs (1989) [28].

Cross-validation was performed to determine the number of latent variables that minimized the predicted residual sum of squares, followed by Van der Voet test (1994) [34] used to identify the simplest model with a not significantly larger error than the absolute minimum.

In validation, the same data set was randomly split into two datasets: two-third of the total data set formed the calibration set and the remaining one-third of the samples, the validation set. This procedure was repeated one hundred times, to verify robustness and stability of the models and then the calculated statistics at each iter were averaged. The performance of PLSR prediction model was evaluated by means of three statistics: (1) the coefficient of determination *R^2^* of regression: observation vs. prediction; (2) root mean square error (RMSE); and 3) the ratio of performance to interquartile distance (RPIQ) [35] defined as follows:

(3) $$R^{2}=1-\frac{\sum_{i=1}^{n}{\left(y_{i}-y_{i}^{\prime }\right)}^{2}}{\sum_{i=1}^{n}{\left(y_{i}-\bar{y}\right)}^{2}}$$

$y_{i}$ is the observed values, $\bar{y}$ is their average, $y_{i}^{\prime }$ the predicted values and n is the number of samples used for validation with *i* = 1, 2, ..., n.

*IQ* is the difference between the third and first quartiles of y:

*IQ* = *Q_3_* − *Q_1_* (4)

and measures the spread of the data. RMSE and *IQ* have the same unit of DNA measurement; therefore, RPIQ is unitless and measures the accuracy of the prediction model [36]. R^2^ measures the proportion of variance of observations explained by the model.

After a preliminary cross-validation test using raw DNA data, the residuals were tested for normality with Shapiro–Wilk and Kolmogorov–Smirnov tests. As such, in case of distribution deviated from the normal one, the DNA data were submitted to normalizing rank transformation [37,38,39]. From now on, all elaborations refer to the normal ranks of DNA data.

Variable Importance in the Projection (VIP) statistic was used for the selection of the relevant wavelengths since it gives some insights on the relative importance of wavelengths to predict DNA content by qPCR analysis [40]. In fact, VIP statistics consider both predictors and response variables, because it is a weighted sum of squares of PLSR X-score coefficients (loadings) for the retained components (latent variables), with the weights calculated from the amount of dependent variable (y) variance explained by each retained component [41]. Since the average of squared VIP scores equals 1 [42,43], a threshold value of 1 was chosen and the wavelengths with a VIP score greater than 1 were considered significant.

Discriminant analysis is a multivariate statistical technique that is commonly used as a powerful classification approach when response variable is categorical: it uses multiple quantitative attributes to discriminate single classification variable. Each observation is placed in the class from which it has the smallest generalized squared distance (*D*) [44], calculated for each group (*j*) according to the following formula:

(5) $$D_{j}^{2}\left(X\right)={\left(X-\bar{X_{j}}\right)}^{\prime }\;{COV}^{-1}\left(X-\bar{X_{j}}\right)$$

where ***X*** is the vector of multivariate observations for a given pixel of the map, $\bar{X_{j}}$ is the vector of the means of variates for the class *j* and *COV* is the covariance matrix [45,46,47].

In the present study, discriminant analysis was used to investigate the radiometric differences between the two categories indicated with the value 0, when *Xfp* was absent, and the value 1 when it was present.

The two procedures, PLSR and discriminant analysis, should not be considered in an alternative but integrated way. The first allowed us to identify those bands that contribute most to the prediction of DNA content, considered as a continuous numerical variable. The discriminant analysis instead allowed us to identify those wavelengths that most contribute to the separation of the two classes previously defined and based on the fixed threshold value of DNA. It, therefore, depended strongly on the criterion used for this classification as well as, similarly to PLSR, on the quality of DNA measurements.

The discriminant analysis consisted of the following six steps:

1. Normalization of DNA data was performed after estimating the skewness and kurtosis with SAS/UNIVARIATE procedure (data not shown). The within-class covariance matrices were pooled because Bartlett test for within-class variance homogeneity was not significant at a level of 0.10.
2. Univariate analysis of variance (ANOVA) was carried out to test the hypothesis that the means of each variable (reflectance) between two categories (0 and 1) were equal. The multivariate analysis of variance (MANOVA) compared multivariate means across several variables (reflectances). Four multivariate statistical tests were used: Wilks’ lambda, Pillai’s trace, Hotelling–Lawley trace, and Roy’s maximum root [45].
3. Canonical Discriminant Analysis (CDA) extracted one linear combination (number of categories [2] minus 1) of the quantitative variables (reflectances) that best revealed the differences between the two categories. The extracted canonical variable had the highest possible multiple correlation with the categories. The standardized coefficients indicated the partial contribution of each variable (reflectance) to the canonical variable and were commonly used to interpret the meaning of the canonical variable.
4. Classification accuracy was assessed using the error matrix calculated in a cross-validation test [48]. The overall accuracy was calculated by summing up the correctly classified observations along the main diagonal of the error matrix. The class-specific error-count estimate was also calculated as the proportion of miss-classified observations in the class.
5. Step Discriminant Analysis (SDA) was also carried out to determine the bands enabling maximum discrimination between the two categories 0 and 1 [49]. SDA was performed with STEPDISC procedure of SAS/STAT using the stepwise algorithm (SAS ANALYTICS U). Significance level for a variable to entry and to stay was set at 0.01.
6. Finally, a K-means clustering method was performed on only spectral data, using SAS/FASTCLUS procedure, by minimizing the sum of squared distances from the cluster means [50]. Unsupervised classification was added to the discriminant analysis (supervised classification) to compare the two categories by confusion matrix and to investigate whether the olive foliar radiometric response may also depend on other factors independent of the presence of the bacterium.

## 3. Results and Discussion

### 3.1. Selection of Optimal Spectral Bands Using Data Set With DNA Content as Response Variable and Spectral Reflectance as Redictors: PLS-VIP Statistics

The descriptive statistics of the leaf DNA content (ng µL^−1^) are reported in Table 1.

The inspection of the table indicated a wide variability in DNA content. Both tests (Shapiro–Wilk and Kolmogorov–Smirnov) verified that DNA data distribution shifted from normal distribution, as shown also by skewness and kurtosis values (Table 1). These parameters, describing the distribution shape, showed that most values were quite like the mean, but with a long tail of few very large values. Such a data distribution suggested the need to submit DNA data to Gaussian transform also to improve the linearity of the relationship with the spectra.

The results of the PLS analysis, after DNA data normal transformation (Table 2), showed that the number of factors extracted with cross-validation was 15, while Van der Voet’s test extracted six significant factors at *p* ≤ 0.10, which explained 96.62% of variance. The goodness of prediction was evaluated by RMSE = 0.53, R^2^ = 0.68, and RPIQ = 2.45. These three statistics, while detecting a good degree of fitting, were not optimal [35]. An explanation might be found in the dis-matching between the small measurement support of the radiometer and the one of the composite samples for PCR measurement, which might have caused an excessive dilution of the DNA content even below the detection limit.

**Figure 2:**
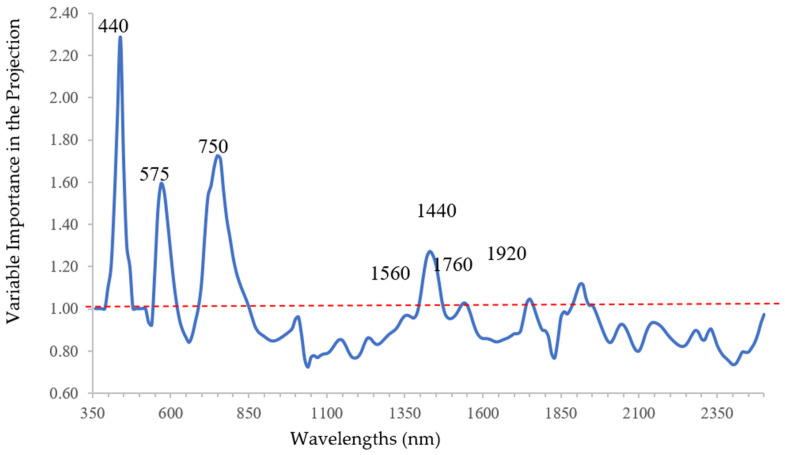
Variable Importance in the Projection (VIP) profile of the selected Partial Least Square Regression (PLSR) model predicting DNA content. Relative maximum peaks are marked.

According to VIP analysis, four main ranges of wavelengths were more powerful in predicting the DNA content: (1) 360–520 nm, with a peak at 440 nm; (2) 550–620 nm, with a peak centered at 575 nm; (3) 690–860 nm with a peak centered at 750 nm; (4) 1390–1490 nm, with a peak at 1440 nm (Figure 2).

These peaks highlighted the relationship between the DNA content and the corresponding wavelengths. The highest peak at 440 nm, considered also in a stress index, as NPQI (Normalized Phaeophitinization Index) [21,22], showed the influence of DNA content on blue reflectance of the surveyed leaves. Similar results were found by Poplete et al. (2020) [51] when asymptomatic and symptomatic leaves sampled on infected trees showed an influence of X*f* induced-stress on blue-reflectance. Zarco-Tejada et al. (2018) [20] found that NPQI was the best index with CSWI, SIF, and Radiative transfer model derived features to distinguish between *X. fastidiosa* infected and uninfected olive trees, also when the latter were assessed in field as asymptomatic by trained plant pathologists. However, it was less efficient to discriminate between initial and advanced stages of the plant disease.

The peak at 575 nm, used in a stress index as photochemical reflectance index (PRI), suitable for estimating photosynthetic light use efficiency [22,52,53], and the peak at 750 nm, already used as a stress vegetation index [54] related to chlorophyll content in indices VOG2 [55], might certainly be associated with an alteration in the chlorophyll function.

The peak at 1440 nm with the other lower peaks at 1560, 1760, and 1920 seemed to show absorption mechanisms that mainly affect functional groups such as the O-H binding of some compounds involved in biofilm matrix during infection process [10]. The absence of peaks in SWIR between 2000–2500 nm seemed to be the confirmation.

In particular, Fourty et al. (1996) attributed the peak at 1760 nm to lignin [56] since the synthesis of this compound in vegetal textures might be associated to a tolerance mechanism with a slow disease progression [57].

### 3.2. Selection of the Optimal Spectral Bands Using Data Set With DNA Categorial Variables and Spectral Reflectance as Predictors: Discrimination of the Two Classes of Olive Plants

#### 3.2.1. Canonical Discriminant Analysis (CDA)

The ANOVA analysis showed that the means of the two groups were significantly different for all spectra, with F test at a probability level of *p* < 0.05 (data not shown).

The MANOVA analysis, performed with different tests, showed a high statistical significance in all cases (Table 3), i.e., the spectral data were quite efficient in discriminating the two categories based on the DNA analyses.

The standardized coefficients of the canonical variable in descending order, selecting only those more weighting (less than 0.05 quantile and greater than 0.95 quantile in the data distribution of the coefficients) are listed in Table 4. Unlike the VIP analysis, the canonical analysis showed a possible spectrum alteration due to the presence of the bacterium preferably in the NIR range (790–1000 nm), and in the initial part of the SWIR range up to 1790 nm, rather than in the visible range. These results seemed to confirm other results ascribable to an evident alteration in foliar water status [19,58].

Comparing the results, the wavelengths resulting from the PLSR did not match with those resulting from the CDA, with one exception regarding wavelengths around 1700 nm, ascribable to lignin used by plants for cellular wall [59,60].

More, the prediction of DNA content, assessed by PLSR, seemed influenced by biochemical compounds with an important hydrophilic component. It might occur because of the bacterial production of the biofilm occluding xylematic vessels [10]. Instead, CDA results seemed to evidence the wavelengths responding to water stress occurring when an uninfected leaf becomes infected, discriminating between the two categories.

Far from being contradictory, the two approaches can be used in an integrative way, adding information to the knowledge on the alteration of the physical-chemical characteristics of the leaves caused by *Xfp*. The differences between the two statistical procedures might be explained by the fact that while PLSR was based on actual measurements of DNA content, discriminant canonical analysis also depended on the classification criterion used: the relevant wavelengths detected were different because PLSR referred to infected leaves, while CDA was able to discriminate the infected leaves by the uninfected ones.

The results of CDA (Table 5) are reported in terms of Receiver Operating Characteristic (ROC) analysis: the model had a true positive rate (TPR) or sensitivity of 0.65, a false positive rate (FPR) of 0.30. The model had an overall accuracy of 0.67 and showed a better discrimination capacity for infected leaves but a quite low sensitivity to uninfected leaves. There are several reasons for this among which one of the most remarkable could be the different support of the two types of measurements. The predictive capabilities of the model would certainly be improved if laboratory measurements were carried out at single leaf scale, instead of the pooled sample.

Nevertheless, the estimated model might be used as a tool to make decisions in planning olive tree sampling. Further plant scouting could be directed towards those plants for which the probability of erroneous classification is greater. Even if preliminary, these results seemed to confirm the prospect of hyperspectral data analysis in discriminating *Xfp*-infected or uninfected olive plants as reported also by other researchers [20].

#### 3.2.2. Stepwise Selection

Stepwise selection highlighted three relevant bands: (1) 530 nm in VIS; (2) 780 nm in NIR; (3) 1360 nm in SWIR1 (Table 6), coherently with the previous results.

The wavelength at 530 nm confirmed the result of VIP analysis as related to chlorophyll function [61].

The band at 780 nm was influenced by anthocyanin pigment [62]. Several abiotic and biotic stresses determine significant accumulation of anthocyanins in the leaves, among which drought and bacterial infections are included, similarly to the conditions induced by *Xfp*-infection [63].

The band at 1360 nm in plant was related to leaf water content as previously reported [58].

Therefore, it seemed that the bacterial infection impaired the main activities of the plant, such as the chlorophyll, the nutrition, and the transpiration functions: this was an expected result. However, the power of radiometric investigation is to detect the spectral anomalies before the appearance of visible symptoms. This is particularly relevant for *Xfp* infections, whose latency period is very long (12–14 months) [15]. In fact, the early detection of infected plants, in advance of symptoms appearance, is crucial for the prompt application of phytosanitary regulations, such as the felling of infected trees, thus, avoiding the spread of the pathogen to the surrounding healthy trees.

### 3.3. Unsupervised Classification Using Data Set of Spectral Data

Clustering analysis identified three significant clusters (Table 7): a very compact cluster 1, with 36 spectral data belonging only to category 1; a mixed cluster 2, with 100 spectral data mostly belonging to the category 1 (82%); and a mixed cluster 3, with 89 spectral data with a prevalence of the category 0 (70.8%). It is worth to note that cluster 1 comprised only spectra form plants classified as asymptomatic, i.e., with no visible disease symptoms, even though resulted infected by qPCR analysis.

The fact that leaves belonging to asymptomatic but infected trees have been grouped in a single cluster, indicated that their spectral responses differed significantly not only from those of non-infected plants, but also from those of infected and symptomatic plants. These results highlighted the potential of hyperspectral radiometer to discriminate plants that might be infected, although visually asymptomatic, thus, indicating the specimens on which the laboratory diagnostic test should be preferably performed. This would allow driving the sampling, thus, reducing the costs, improving the efficiency of the monitoring procedure for quarantine pathogens.

However, the fact that clustering with only radiometric data has led to the production of not completely compact but mixed groups, induced us to think, as expected, that the radiometric response of a plant was the result of multiple interacting external and internal factors. Certainly, the presence of the bacterium caused an alteration in the physiological functions of the plant, which influenced its spectral signature, but these alterations could not be univocally assigned to a specific infection.

## 4. Conclusions

Hyperspectral data were analyzed with a set of integrative statistical techniques, to evaluate their potential in detecting olive plants infected by *Xfp*. The different methods produced consistent and integrable results for a more comprehensive understanding of the phenomenon under investigation.

When spectral data were analyzed as predictors of bacterial DNA content, using PLSR, the bands related to chlorophyll function were the most relevant. As regards to the discrimination between infected and uninfected plants, previously classified, CDA and stepwise added other bands related to water content and lignin, since desiccation is a clear symptom of *Xfp* infection. These results emphasize the usefulness of using multiple statistical techniques, not in an alternative but in a complementary way.

The confusion matrix for CDA showed that the model had a good overall accuracy of 0.67, but with a better capability to discriminate infected plants as compared to the uninfected ones. This not excellent result was probably due to the use of a composite sample for real time-PCR analysis. In this regard, it should be advisable to run qPCR measurements on a single leaf, thus, improving the matching between radiometric and laboratory measurement supports.

Unsupervised classification, using the spectral data only, was able to discriminate the infected plants at a very early stage of infection or even asymptomatic, on which sampling/monitoring should be preferably directed, thus, saving time and financial resources. However, the analyses also highlighted the limitations of the hyperspectral radiometer sensor, which cannot be used as the unique diagnostic tool, even after accurate calibration, due to the plethora of factors that might produce similar symptoms. With the statistical analyses used, the selected wavelengths were associated to biological functions, thus, confirming the results from other works.

An interesting prospect for future research would be to verify if the same results could be obtained by analyzing both full hyperspectral spectra and discrete spectra, including only the wavelengths identified by discriminant analysis. This would make it possible to design lightweight and efficient hyperspectral radiometers to be used on board drones for a preventive survey of olive groves. Unfortunately, it cannot be established a priori, given the multitude of factors that can affect spectral signatures and are not uniquely linked to *Xfp* infection. Nevertheless, one of the most significant results of this research is marking out paths for future research.

Finally, an integrated system of statistical techniques for the processing of radiometric data has also been proposed, that could be used as decision support for planning an efficient and cost-effective sampling/monitoring scheme of the quarantine pathogens. Given the complexity of the phenomenon under study, an interdisciplinary approach including in the research team statisticians, experts in imaging analysis, plant pathologists, epidemiologist, and agronomists, should be strongly encouraged.

## Figures and Tables

**Table 1:** Basic statistics of the DNA content and normality test results (Shapiro–Wilk and Kolmogorov–Smirnov).

	DNA ng µL^−1^
Mean	0.11
Standard Deviation	0.39
Minimum	0.00
Maximum	1.73
Skewness	3.68
Kurtosis	12.99
Shapiro–Wilk (W)	0.34
Probability < W	0.0001
Kolmogorov–Smirnov (D)	0.499
Probability > D	<0.010

**Table 2:** Statistics of Partial Least Square (PLS) by pre-processing method, analysis of residuals (normality tests).

Minimum Root Mean PRESS	Number of Extracted Factors ^a^		Percent Variation Accounted for by PLS Factors
			Model Effects (X-Scores)
0.66	15	6	57.69
			14.93
			7.35
			0.95
			14.00
			1.70
			96.62

^a^ Factors extracted with cross-validation and the smallest number (italics) of significant factors at *p* ≤ 0.10 according to the van der Voet test.

**Table 3:** The MANOVA results regarding the classification criteria.

Statistic	Value	F Value	Pr > F
Wilks’s lambda	0.0026	17.85	<0.0001
Pillai’s trace	0.9974	17.85	<0.0001
Hotelling–Lawley’s trace	382.01	17.85	<0.0001
Roy’s greatest root	382.01	17.85	<0.0001

**Table 4:** Estimates of total-sample standardized coefficients of the canonical variable.

Wavelengths	Total-Sample Standardized Coefficients of the Canonical Variable
980	3.74
790	3.21
1050	3.06
1170	2.90
1100	2.85
1720	2.83
1650	2.61
930	2.14
1290	2.10
910	1.98
1780	−2.07
1730	−2.08
730	−2.32
1230	−2.43
800	−2.56
1040	−2.82
1660	−2.91
990	−3.05
920	−3.37
1110	−3.46

**Table 5:** Confusion matrix deriving from Canonical Discriminant Analysis (CDA) applied on categorial data set (0, uninfected; 1, infected).

		Observed		
		Positive Condition	Negative Condition	
Predicted	positive condition	T1 94	F1 24	118
	negative condition	F0 50	T0 57	107
	Total population	144	81	225

T1 (True positive); F1 (False positive); T0 (True negative); F0 (False negative); TPR (True positive rate or Sensitivity) = ΣTrue1/Σpositive condition = 0.65; FPR (False positive rate) = ΣFalse1/Σnegative condition = 0.30; PPV (Positive Predicted Value or Precision) = ΣTrue1/Σpredicted condition positive = 0.80; ACC (overall Accuracy) = (ΣTrue1+ΣTrue0)/Σtotal population = 0.67

**Table 6:** Results of stepwise selection carried out on DNA categories and reflectance data *X* in the range of 350–2500 nm.

*X* Variables	Average Square Canonic Correlation	F Value	Pr > F
780	0.42	161.90	<0.0001
1360	0.48	23.20	<0.0001
530	0.58	35.68	<0.0001

**Table 7:** Results of unsupervised classification applied to spectral data.

Cluster	Frequency			
	0		1	
	Asymptomatic	Symptomatic	Asymptomatic	Symptomatic
1	0	0	36	0
	0.0 *	0.0 *	100.0 *	0.0 *
2	18	0	49	33
	18.0 *	0.0 *	49.0 *	33.0 *
3	45	18	14	12
	50.6 *	20.2 *	15.7 *	13.5 *

* percentage of leaves belonging to asymptomatic and symptomatic plants within every cluster (1,2,3) for each category (0 and 1).

## Data Availability

Data is contained within the article.
